# Do girls with depressive symptoms exhibit more physical aggression than boys? A cross sectional study in a national adolescent sample

**DOI:** 10.1186/s13034-015-0064-5

**Published:** 2015-08-22

**Authors:** Xavier Benarous, Christine Hassler, Bruno Falissard, Angèle Consoli, David Cohen

**Affiliations:** Department of Child and Adolescent Psychiatry, Hôpital Pitié-Salpêtrière, 47-83, Boulevard de l’Hôpital, 75013 Paris, France; Inserm U669, PSIGIAM, Maison des Adolescents, 97 Boulevard de Port Royal, 75679 Paris Cedex 14, France; 19 rue de Turenne, 75004 Paris, France

**Keywords:** Adolescents, Aggressive behaviors, Depression, Physical aggression, Gender paradox

## Abstract

**Background:**

The relationship between depression and aggressive behaviors in adolescents has previously been reported in clinical and epidemiological studies. However, there is conflicting evidence concerning the effect of gender on this relationship. This study tested whether the link between depressive symptoms and physical aggression differed between boys and girls in a large community-based sample of adolescents.

**Methods:**

A cross-sectional sample of adolescents aged 15–19 (N = 6,677) was studied within the 2007 ESPAD national survey. Depressive symptoms were assessed using the Adolescent Depression Rating Scale. We distinguished adolescents with subthreshold levels of depressive symptoms and adolescents with clinically significant levels of depressive symptoms. Physical aggressive behaviors in the last year were reported using items from the Antisocial Behavior Scale.

**Results:**

After adjusting for confounding variables, the odds-ratio between depressive symptoms and physical aggressive behaviors was around 1.4. This relationship was stronger for girls than for boys in presence of clinically significant levels of depressive symptoms, but did not differ between the genders in the case of subthreshold levels of depressive symptoms.

**Conclusions:**

Girls with severe depressive symptoms were more likely to present physical aggressive behaviors than boys. Future studies will be needed to explore the role of irritability in these differences.

## Background

In contrast to adults, the clinical presentation of depressive disorder in adolescence frequently involves irritable mood and externalized symptoms [1]. In addition, girls who present both depressive symptoms and aggressive behaviors are substantially more impaired than their male counterparts [2–4]. Nevertheless, there is relatively little research on how far the relationships between mood symptoms and aggressive behaviors differ between males and females. In this paper, we examine this issue in a large, self-report, cross-sectional study in a community-based sample.

The relationship between depressive symptoms and aggressive behaviors has previously been reported in clinical [5–8], and epidemiological studies [9–11]. This association is important to consider for clinicians, as aggressive behavior may hide core thymic symptoms [1, 12–14], and excessive focus on external behaviors can then lead to a misdiagnosis of depressive disorder at this age. This is particularly regrettable given the evidence that early recognition and treatment could limit functional impairment [12]. In addition, girls with depressive disorder associated with behavioral problems (including aggression) appear to present poorer outcomes compared to boys, with a higher mortality rate [15], poorer health [15], more frequent comorbid psychiatric disorders [16] and a higher suicide rate [4].

From early adolescence, the gender-ratio for depression increases greatly (reaching 2/1 for girls) [17]. It has also been suggested that depressive episodes could differ in their presentation between genders. In particular, irritability, which is a core symptom of depression in young people, appears to be more common in females than in males [18]. As irritable mood is characterized by excessive reactivity to negative emotional stimuli, irritable individuals are more likely to be angry or aggressive in response to provocation [19]. Considering the higher level of irritability in depressed females compared to males, it could be expected that depressed female adolescents might exhibit more aggressive behaviors.

This hypothesis is supported by contrasting evidence. Wiesner and Kim reported that among adolescents with depressive symptoms, more girls than boys engaged in high levels of delinquent behavior over a 2-year period [20]. In the Great Smoky Mountains study, Stringaris et al. noted that girls with depressed and irritable mood were more likely to develop a conduct disorder than boys [21]. In contrast to this, Chen and Simons-Morton noted that among adolescents with high levels of depression, more boys than girls were in the highest trajectory for conduct problems over a 3-year period (from Grade 6 to Grade 9) in a community sample [22].

It has been suggested that such disparities could partly result from the different definitions of aggressive behaviors used [4]. Aggressive behaviors are traditionally distinguished from one another by determining the form of aggression [19]. Physical aggression is distinct from other types of aggression (e.g. verbal aggression) for its specific developmental trajectory and its association with psychopathology [23]. A large number of studies have stressed the relationship between physical aggression and emotional disorders in young people, compared to other forms of antisocial behaviors [24]. In line with previous studies, we therefore specifically focused on physical aggression in this research.

Gender differences in the association between depressive symptoms and aggressive behaviors may result from a particular association with a third factor. For example, the stronger association hypothesised between aggressive behaviors and depressive symptoms in females than in males could be partly explained by the distinct patterns of substance use and abuse between genders observed in adolescents [25–27]. Indeed, substance use has been associated with both emotional disorders and conduct disorders in adolescents in a community-based study [27]. It is worth taking into account the possible role of environmental risks factors for both aggressive behaviors and depressive disorder, especially those for which males and females differ (for instance exposure to adverse life events) [16, 28, 29].

We first tested the hypothesis that the prevalence of physical aggressive behaviors would be markedly higher in adolescents who were depressed compared to those who were not. This was based on epidemiological studies, where a high level of association between depression and aggressive behaviors in adolescence has been reported.

Secondly, we sought to compare the relationship between depression and physical aggressive behaviors in males and females. Previous studies have suggested that the exacerbation of aggressive behaviors in depressed young people is partly mediated by irritability [5]. As irritability is more frequently reported in depressed girls than in boys, we expected to find a stronger relationship for girls than for boys.

## Methods

### Sample

Data were collected as part of the 2007 European School Survey Project on Alcohol and other Drugs (ESPAD). A national sample of 8,843 French secondary school students was formed and data was collected using an anonymous, self-administered questionnaire completed in a classroom setting between April and May 2007. The participation rate for schools was 98%, and 90% of the pupils participated in the survey. The ESPAD study covered the whole of secondary school. The Ministry of Education conducts a population census of the population of pupils each year in September. The sample of schools was drawn from a computerized list of 404 schools, in a stratified random sample of schools proportionate to school size. Two classes were selected in each school. The stratification took into account the type of school, the type of area (urban/rural) and the educational characteristics. The sample was considered to be self-weighted. Participation in the ESPAD survey was voluntary and anonymous following passive parental consent. Pupils completed the self-administered questionnaire in about 45 min. The preferred sampling method was the random selection of classes with equal probabilities from comprehensive lists, followed by completion of the self-administrated survey questionnaire by all of the pupils from the selected classes who were present on the day of the data collection. The investigation was approved by an ethics committee (the *Commission Nationale Informatique et Liberté*) and written informed consent was obtained from respondents. Details of sampling, survey methods and other information, including response rates, can be found in Hibell [30].

### Instruments

From the ESPAD questionnaire, we used the data available about aggressive behaviors, the ADRS score, and data from the psychosocial module.

Aggressive behaviors were measured using the Antisocial Behavior Scale as part of the 2007 ESPAD Psychosocial Module. It was taken from the Monitoring the Future Survey conducted in the USA [31]. The scale features ten items relating to behavioral problems in the last 12 months. Answers are given on a five-point Likert scale ranging from “0” to “5 times or more”. All answers were dichotomized to facilitate analyses, which was possible on account of the U-shape of the distribution of answers, with few intermediate responses. The value of Cronbach’s α was 0.90. Using Principal Component Analysis (PCA) two groups of items were identified: (1) items “b” (taken part in a fight) and “e” (started a fight with another individual); (2) the items “d”, “f”, “g” (referring to theft), “c” (used any kind of weapon to get something from a person) and “h” (damaged public or private property on purpose). The presence of physical aggressive behaviors was based on one positive answer to “b” or “e” (Cronbach α = 0.66). The presence of other antisocial behaviors was based on one positive answer to “d” or “f” or “g” or “c” or “h” (Cronbach α = 0.73).

Current depressive symptoms were assessed using the Adolescent Depression Rating Scale (ADRS) [27]. This scale has been validated on young people aged 12–20. It is a 10-item, self-administered questionnaire with True/False responses concerning the 2 weeks preceding completion. We used the cut-off suggested by Revah-Levy et al. [32]. The sum of the item scores provides a score that divides the population into three distinct groups: “no depressive symptoms” for a score of 0–4, “subthreshold levels of depressive symptoms” for a score of 4 or 5, and “clinically significant levels of depressive symptoms” for a score of 6 or more.

Other variables potentially affecting adolescent behavior were also considered. These were: alcohol consumption in the previous month (1 = 10 or more times, 0 = less than 10 times), lifetime use of cannabis (1 = once or more, 0 = never), lifetime sexual abuse (1 = once or more, 0 = never), school under-attainment (repeated school years, 1 = once or more, 0 = never), maternal educational level (1 = baccalaureate or more, 0 = less than baccalaureate) and single-parent family (1 = yes, 0 = no). Adjusting for confounding variables is important, as previous studies report an association between depression and alcohol use, cannabis use [25–27], traumatic events (e.g. sexual abuse) [16], low perceived family support [28], a low level of maternal education [29], and under-attainment at school [12].

### Analysis

Descriptive analyses of the main socio-demographic characteristics of the sample were performed using Chi squared tests to compare qualitative variables, and Student tests for quantitative variables. A difference was considered as significant at *p* < 0.05. For Chi square analyses Cramer’s phi (φ) effect sizes were calculated.

The first question was whether the prevalence of physical aggressive behaviors differed between adolescents with depressive symptoms and those without. The frequencies of physical aggressive behaviors were compared between the group with ADRS score <4 and the group with ADRS score ≥4, by means of a Chi squared test. We hypothesised that adolescents in the latter group would be more likely to present physical aggressive behaviors than those with a score <4. Then, to control for possible confounders in the relationship between depressive score and physical aggressive behaviors, a multiple linear regression was performed. The dependant variable was the level of depressive symptoms (i.e. the ADRS score). The independent variable was the presence of physical aggressive behaviors. We included the covariates: age, gender, alcohol consumption in the previous month, lifetime use of cannabis, lifetime sexual abuse, repeated school years, maternal educational level and single-parent family. To measure specifically the association between depressive symptoms and physical aggression, but not other antisocial behaviors (e.g. theft), this model was adjusted on the variable “Other antisocial behaviors”. A similar analysis was performed with depressive symptoms operationalized as a categorical variable (i.e. the presence of a clinically significant level of depressive symptoms).

The second question in this paper was whether the link between depressive symptoms and physical aggressive behaviors would differ between male and female adolescents. We ran the same models as presented in the previous paragraph with an interaction term between gender and physical aggression. Then, we compared the results from the model run separately for boys and girls (in this case, we included neither the covariate gender, nor the interaction term). To provide additional support that the gender difference in the relation between depressive symptoms and physical aggressive behaviors was specific to physical aggression and not to antisocial behavior in general, we explored whether the association between depressive symptoms and other antisocial behaviors differed by genders.

## Results

### Description of the sample

Table 1 lists the socio-demographic characteristics of the sample (*N* = 6,677). The survey included 51% girls and 49% boys. The mean age was 16.2 years, SD = 0.8. For depressive symptoms, 8% of the adolescents had clinically significant levels of depressive symptoms, 11% of the girls and 6% of the boys, *χ*^2^ (1, *N* = 6,677) = 68.86, *p* < 0.001, φ = 0.15. Around 20% had subthreshold levels of depressive symptoms, 25% of the girls, and 15% of the boys, *χ*^2^ (1, *N* = 5,774) = 126.20, *p* < 0.001, φ = 0.15. Physical aggressive behaviors were reported by 42% of the adolescents, 33% of the girls and 51% of the boys, *χ*^2^ (1, *N* = 6,486) = 323.33, *p* < 0.001, φ = 0.22. Other antisocial behaviors were reported by 31% of the adolescents, 28% of the girls and 34% of the boys, *χ*^2^ (1, *N* = 6,677) = 168.98, *p* < 0.001, φ = 0.16.

**Table 1:** Socio-demographic and clinical characteristics of the sample

	Total		Girls		Boys	
	N = 6,677	100%	N = 3,402	51.0%	N = 3,275	49.0%
Age	16.27 ± 0.83		16.29 ± 0.84		16.24 ± 0.83	
Absenteeism	1,139	17.5	557	16.4	582	17.8
Repeat school year	2,837	42.5	1,399	41.1	1,438	43.9
Lifetime sexual abuse	406	6.1	311	9.1	95	2.9
Family situation						
Mother’s educational level (at least Baccalauréat)	3,069	46.0	1,500	44.1	1,569	47.9
Single-parent family	996	14.9	545	16.0	451	13.8
Substance use						
Alcohol consumption (≥10 times per month)	910	13.6	317	9.3	593	18.1
Cannabis use (at least one in lifetime)	2,278	34.1	1,054	31.0	1,224	37.4
Depressive symptoms						
Mean score	4.29 ± 3.26		4.74 ± 3.20		3.83 ± 3.27	
No depressive symptoms (ADRS score <4)	4,441	66.5	2,036	59.8	2,405	73.4
Subthreshold level of depressive symptoms (ADRS score = 4,5])	1,333	20.0	845	24.8	488	14.9
Clinically significant level of depressive symptoms (ADRS ≥6)	572	8.4	388	11.4	184	5.6
Aggressive behaviors						
Physical aggressive behaviors	2,278	34.1	829	24.4	1,449	44.2
Other antisocial behaviors	6,102	91.4	3,258	95.8	2,844	86.8

### Depressive symptoms and physical aggressive behaviors

As hypothesised, adolescents with depressive symptoms reported higher rates of physical aggressive behaviors (53%) compared to those without depressive symptoms (38%), *χ*^2^ (1, *N* = 6,289) = 86.11, *p* = 0.001. Depressive symptoms had a small effect on the presence of physical aggressive behaviors in adolescents, φ = 0.12, OR = 1.69 [1.51–1.89]. The relationship between physical aggression and the level of depressive symptoms remained significant after adjusting on covariates (Table 2)

**Table 2:** Association between depressive symptoms and aggressive behaviors

Outcome as a continuous variable	Predictive variables	*β*	95% CI	*p*
ADRS score	Physical aggressive behaviors	0.802	0.645; 0.958	0.000
	Other antisocial behaviors	−1.365	−1.669; −1.062	0.000
	Gender	−1.260	−1.409; −1.111	0.000
	Age	−0.172	−0.265; −0.080	0.000
	Alcohol consumption	0.064	−0.082; 0.211	0.391
	Cannabis use	0.333	0.162; 0.504	0.000
	Lifetime sexual abuse	1.474	1.262; 1.685	0.000
	Repeat school year	0.618	0.464; 0.771	0.000
	Single-parent family	0.141	0.031; 0.251	0.012
	Educational level of mother	−0.107	−0.105; 0.083	0.824

Outcome as a categorical variable	Predictive variables	OR	95% CI	*p*
Clinically significant levels of depressive symptoms	Physical aggressive behaviors	1.45	1.14; 1.84	0.002
	Other antisocial behaviors	0.36	0.24; 0.54	0.000
	Gender	0.38	0.30; 0.48	0.000
	Age	0.90	0.78; 1.04	0.169
	Alcohol consumption	0.96	0.73; 1.21	0.735
	Cannabis use	1.25	0.96; 1.61	0.095
	Lifetime sexual abuse	0.93	0.64; 1.35	0.694
	Repeat school year	1.38	1.08; 1.76	0.009
	Single-parent family	1.10	0.93; 1.30	0.256
	Educational level of mother	0.90	0.78; 1.04	0.170

*β* unstandardized regression coefficient, *OR* odds-ratio, *CI* 95% confidence interval

Gender (0 = girls, 1 = boys); alcohol consumption (1 = 10 + times in the past month), lifetime use of cannabis (1 = once or more in their lifetime, 0 = never), lifetime sexual abuse (1 = once or more in their lifetime, 0 = never), repeated school years (1 = once or more in their life time, 0 = never), maternal educational level (1 = baccalaureate or more, 0 = less than baccalaureate) and single-parent family (1 = yes, 0 = no)

.

### Differences between males and females in the association

**Fig. 1:**
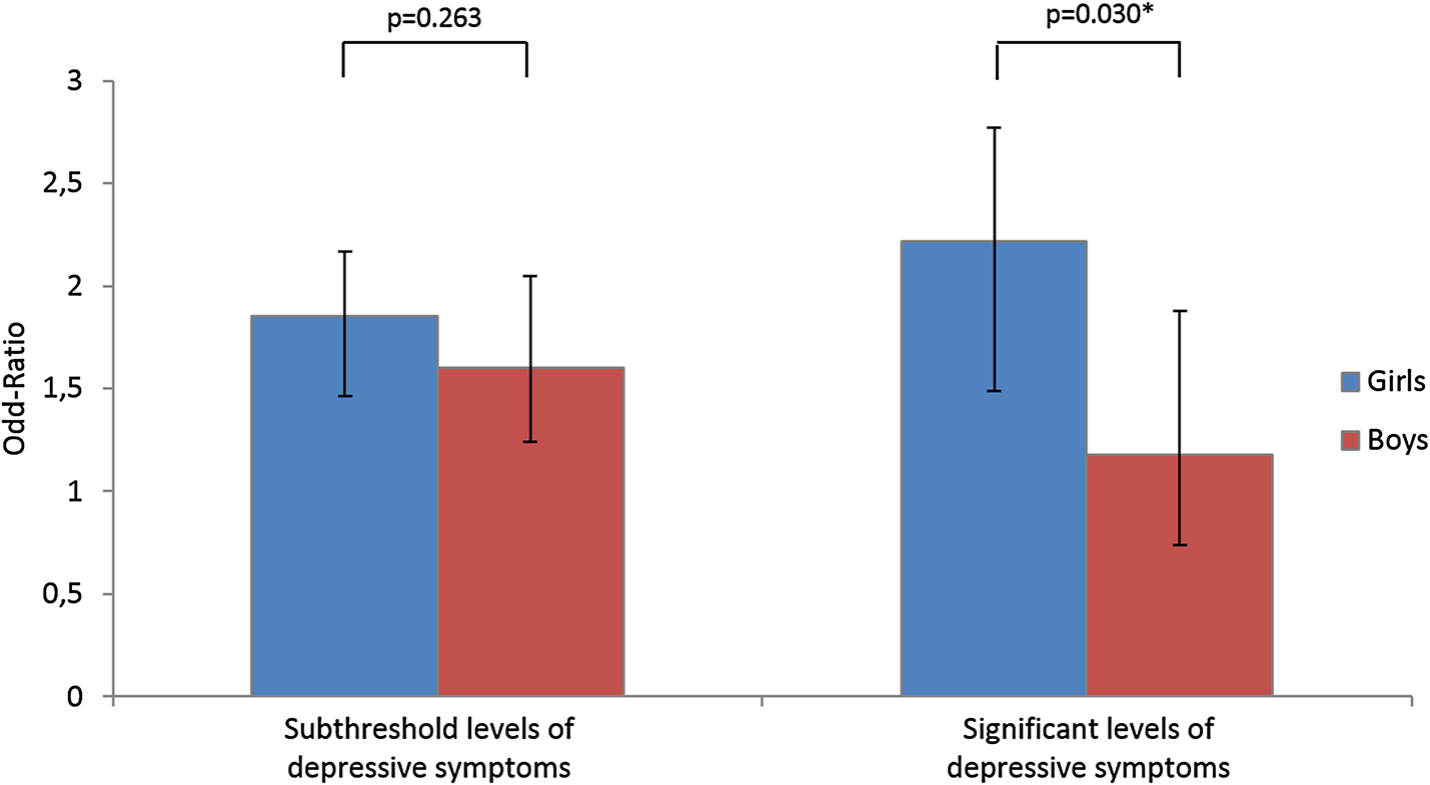
Effect of gender on the relationship between the presence of depressive symptoms and physical aggressive behaviors. *p < 0.05 Results from logistic regression models with the presence of depressive symptoms as the dependant variable and physical aggressive behaviors as the independent variable. Models were adjusted on age, alcohol use, cannabis use, repeat school years, sexual abuse, mother’s educational level, family situation and the presence of other antisocial behaviors

Figure 1 presents the association between physical aggression and significant levels of depressive symptoms in girls and boys. An interaction effect between physical aggressive behaviors and gender was noted, *p* = 0.030. Wald test for the interaction term was *F*(1, 6,665) = 32,765.06, *p* < 0.001. The relationship between physical aggression and significant levels of depressive symptoms was 1.8 times stronger in girls than in boys; OR = 2.22 [1.67–2.95] vs. OR = 1.18 [0.74–1.88] respectively, *p* = 0.030. By contrast, the association between significant levels of depressive symptoms and the variable “other antisocial behaviors” did not significantly differ by gender, OR = 0.30 [0.15; 0.59], *p* < 0.001 for girls; and OR = 0.34 [0.20; 0.56], *p* < 0.001 for boys. When added to the multiple logistic regression, an interaction term between other antisocial behaviors and gender was not found statistically significant, (*p* = 0.239).

### Differences in symptom severity

The frequency of physical aggressive behaviors was higher among the subjects with subthreshold levels of depressive symptoms (50%), compared to those without depressive symptoms (38%), *χ*^2^ (1, *N* = 5,724) = 45.21, *p* < 0.001, φ = 0.09, OR = 1.54 [1.36–1.75]. The interaction term between physical aggressive behaviors and gender was not significant, *p* = 0.469. This association did not significantly differ between males and females: OR = 1.60 [1.24–2.05] vs. 1.85 [1.53–2.24] respectively, *p* = 0.263 (Fig. 1).

## Discussion

This study assessed the relationships between depressive symptoms, physical aggressive behaviors and gender in a large, representative, community-based sample of adolescents aged 15–19 (N = 6,677), adjusting for confounding variables. Depressive symptoms and physical aggressive behaviors were positively and significantly associated, with an OR of around 1.45. This link was stronger in girls than in boys.

The result supports the first hypothesis of a specific relationship between physical aggression and depressive symptoms in adolescents. Indeed, previous studies noted that adolescents engaging in physical aggressive behaviors more frequently present emotional disorders [7, 8, 23, 24]. Irritability may play a key role in this relationship [7, 21, 33, 34].

A stronger association between physical aggressive behaviors and depressive symptoms was observed in girls than in boys. A similar pattern of results was obtained when symptom scores instead of categories were used to compare the two groups. Interestingly, such difference between boys and girls with depressive symptoms specifically concerned physical aggressive behaviors, but not other antisocial behaviors.

This finding appears to confirm that depressive symptoms have gender-specific features in adolescents. For example, a previously reported higher level of irritability in girls than in boys presenting with depressive symptoms could foster impulsive behaviors, and thus physical aggression [1, 7, 21]. If this is true, one could expect that a higher rate of impulsive behaviors would be reported among young depressed females than young depressed males. Indeed, a higher prevalence of deliberate self-harm and suicide attempts has been noted among depressed female adolescents as opposed to males [3, 35]. A higher level of impulsiveness may be underpinned by more marked disturbances of inhibitory control mechanisms during depression in females than in males. Interestingly, previous studies have suggested that depressed boys are more likely to present psychomotor retardation than depressed girls [18, 36]. Alternatively, these results might be explained by the effect of factors common to both physical aggressive behavior and depressive symptoms, which affect boys and girls differently. For example, Compton et al. [37] noted that a poor familial environment (e.g. coercive family interactions) is a shared risk factor for externalized behaviors and depressive symptoms in girls, but only for externalised symptoms in boys [37]. However, in this study, controlling for a whole range of environmental factors did not alter the difference observed in the association between depressive symptoms and physical aggressive behaviors in males and females.

Our findings also contribute to casting light on the gender differences in adolescents with conduct disorders. Indeed, despite a lower prevalence, adolescent girls with conduct disorders are more likely to present emotional difficulties and marked functional impairment in comparison to boys [2–4, 20]. This observation is referred to as a “gender paradox” [2]. On the basis of these findings, the greater impairment of girls with a conduct disorder could partly be explained by a stronger association with depressive symptoms, which could decrease the overall level of functioning. However, testing such a hypothesis would require appropriate measures of conduct disorder and subtypes of aggressive behaviors.

The strengths of the ESPAD survey were its extensive national coverage and its careful methodological standardization. Depressive symptoms were measured with a specific scale for adolescents. Because of the good statistical power, we were able to run multivariate analyses on each gender. In addition, as the study is a community-based sample, this prevents the problems of referral bias or clinician bias, which would tend to overestimate the association between depression and behavioral problems [38].

This study also presented limitations. Firstly, aggressive behaviors were evaluated by grouping items from the Antisocial Behavior Scale in an exploratory approach. The lack of a specific scale to describe each type of aggressive behavior could explain the moderate internal consistency of these measures (α = 0.66 for physical aggressive behaviors). It has also been suggested that self-report could underestimate externalized symptoms in young people [1]. However, Brener et al. noted that anonymous self-reports in the Youth Risk Behavior Survey questionnaire (which contains similar questions to the Antisocial Behavior Scale) had good validity in terms of measuring aggressive and antisocial behaviors in adolescents [39]. Secondly, the design of this study does not provide information about the direction of the association between depressive symptoms and physical aggressive behaviors. Thirdly, our findings are school-based and cannot be extended to adolescents not attending school. It should be noted, however, that the vast majority of adolescents of this age are still enrolled in the school system in France. In the same vein, these findings should not be generalized to younger adolescents (i.e. below the age of 15) without prior replication within this age group. Finally, it was not possible to assess other potentially relevant confounders between depression and physical aggressive behaviors. In particular, parental psychopathology (e.g. depressive disorder and personality disorder) [10, 11], parental alcohol or substance abuse [40] and the presence of anxiety disorders [16] were not assessed.

## Conclusions

Using a large, community-based survey, we found a significant link between depressive symptoms and physical aggressive behaviors. The stronger relationship for girls than for boys could guide future research. Future studies should explore whether depressed adolescent girls have a higher risk of other impulsive behaviors in comparison to boys, and, if this is the case, whether irritability mediates these relationships. This study found that the relationship between physical aggression and depressive symptoms is stronger among adolescent girls than adolescent boys. These findings suggest that clinicians should particularly focus on mood symptoms in adolescent girls who present physical aggressive behaviors.

## Abbreviations

ESPAD: European School Survey Project on Alcohol and other Drugs
ADRS: Adolescent Depression Rating Scale
