# Erratum to: Do girls with depressive symptoms exhibit more physical aggression than boys? A cross sectional study in a national adolescent sample

**DOI:** 10.1186/s13034-015-0085-0

**Published:** 2015-11-23

**Authors:** Xavier Benarous, Christine Hassler, Bruno Falissard, Angèle Consoli, David Cohen

**Affiliations:** Department of Child and Adolescent Psychiatry, Hôpital Pitié-Salpêtrière, 47-83, Boulevard de l’Hôpital, 75013 Paris, France; Inserm U669, PSIGIAM, Maison des Adolescents, 97 Boulevard de Port Royal, 75679 Paris Cedex 14, France; 19 rue de Turenne, 75004 Paris, France

## Erratum to: Child Adolesc Psychiatry Ment Health (2015) 9:41 DOI 10.1186/s13034-015-0064-5

Following publication of this article [1], the authors noticed that the text contained some statistical errors.

The below text

“Physical aggressive behaviors were reported by 42 % of the adolescents, 33 % of the girls and 51 % of the boys, χ^2^ (1, N = 6,486) = 323.33, p

Other antisocial behaviors were reported by 31 % of the adolescents, 28 % of the girls and 34 % of the boys, χ^2^ (1, N = 6,677) = 168.98, p ”

Should be corrected as follows:

“Physical aggressive behaviors were reported by 34 % of the adolescents, 25 % of the girls and 44 % of the boys, χ^2^ (1, N = 6,486) = 323.33, p

Other antisocial behaviors were reported by 91 % of the adolescents, 96 % of the girls and 87 % of the boys, χ^2^ (1, N = 6,677) = 168.98, p ”
